# Factors affecting the community’s attitude toward COVID-19 vaccination: cross-sectional study

**DOI:** 10.1590/0034-7167-2022-0597

**Published:** 2023-12-04

**Authors:** Chintia Gracelia Amalo, Ezra Amarya Ekaristy, Maretty Wattileo, Martina Pakpahan, Ester Silitonga

**Affiliations:** I Universitas Pelita Harapan, Faculty of Nursing. Tangerang, Indonesia; II Siloam Training Center. Tangerang, Indonesia

**Keywords:** Attitude to Health, COVID-19 Vaccines, Vaccination Hesitancy, Vaccination Coverage, COVID-19, Atitude Frente a Saúde, Vacinas Contra COVID-19, Hesitação Vacinacional, Cobertura Vacinal, COVID-19, Actitud Frente a la Salud, Vacunas COVID-19, Vacilación a la Vacunación, Cobertura de Vacunación, COVID-19

## Abstract

**Objective:**

The study aimed to analyze the factors that affect the community’s attitude towards COVID-19 vaccination in Tangerang District.

**Methods:**

A cross-sectional study was used. Convenience sampling was used to select 400 respondents. Inclusion criteria are living in Tangerang District, aged between 18 and 55, and earning a living. An online questionnaire was used and passed validity and reliability tests. This study received ethical approval.

**Results:**

Most respondents had a high level of education (48.50%), low income (72.50%), high knowledge (78%), and a positive attitude (76.50%) regarding vaccination against COVID-19. The Chi-square test revealed a correlation between knowledge and attitudes towards COVID-19 vaccination (p=0.001), as well as education levels (p=0.001), but there was no correlation between income and attitudes (p=0.094).

**Conclusions:**

Health professionals should engage in extensive socialization and face-to-face visits with people with limited access to information to promote a positive attitude and expand the scope of COVID-19 vaccination.

## RESUMO

**Objetivo:**

o objetivo deste estudo foi analisar os fatores que afetam a atitude da comunidade em relação à vacinação contra COVID-19 no distrito de Tangerang.

**Métodos:**

Estudo transversal com uso da amostragem por conveniência para selecionar 400 respondentes. Os critérios de inclusão foram residir no distrito de Tangerang, idade entre 18 e 55 anos e trabalhar. O questionário online utilizado foi aprovado nos testes de validade e confiabilidade. O estudo recebeu aprovação ética.

**Resultados:**

A maioria dos entrevistados possuía alta escolaridade (48,50%), baixa renda (72,50%), alto conhecimento (78%) e atitude positiva (76,50%) em relação à vacinação contra a COVID-19. O teste do qui-quadrado revelou correlação entre conhecimento e atitudes em relação à vacinação contra COVID-19 (p=0,001) e níveis de escolaridade (p=0,001). Não houve correlação entre renda e atitudes (p=0,094).

**Conclusões:**

Os profissionais de saúde devem realizar ampla socialização e visitas presenciais com indivíduos que possuem acesso limitado a informações para promover uma atitude positiva e expandir o escopo da vacinação contra a COVID-19.

## RESUMEN

**Objetivo:**

El objetivo de este estudio fue analizar los factores que afectan la actitud de la comunidad hacia la vacunación contra el COVID-19 en el distrito de Tangerang.

**Métodos:**

Estudio transversal en el que se utilizó un muestreo por conveniencia para seleccionar a 400 encuestados. Los criterios de inclusión fueron vivir en el distrito de Tangerang, edad 18-55 años y trabajar. Se utilizó un cuestionario en línea, el cual pasó pruebas de validez y confiabilidad. Este estudio recibió aprobación ética.

**Resultados:**

La mayoría de los encuestados tenía un alto nivel de educación (48,50%), bajos ingresos (72,50%), alto conocimiento (78%) y una actitud positiva (76,50%) respecto a la vacunación contra el COVID-19. La prueba chi-cuadrado reveló una correlación entre el conocimiento y las actitudes hacia la vacunación contra el COVID-19 (p=0,001) y los niveles educativos (p=0,001). No hubo correlación entre los ingresos y las actitudes (p=0,094).

**Conclusiones:**

Los profesionales de la salud deben realizar una amplia socialización y visitas cara a cara con personas con acceso limitado a la información para promover una actitud positiva y ampliar el alcance de la vacunación contra el COVID-19.

## INTRODUCTION

For about two years, the entire society has been plagued by the Coronavirus Virus Disease (COVID)-19 pandemic. According to the World Health Organization, the number of deaths caused by COVID-19 in 2020-2021 reached 16.6 million^(1)^. Southeast Asia, Europe, and America contributed the most deaths (84%) while lower middle-income countries accounted for around 53% of deaths^(1)^. This suggests that countries should invest in more resilient health systems to maintain essential health services during crises. Herd immunity is a population that has developed immunity to infectious diseases through vaccination.

COVID-19 is transmitted through droplets when talking, coughing, or sneezing at 3-6 feet (1-2 meters), herd immunity requires that most of the population receive COVID-19 vaccination as the most effective method of preventing COVID-19 spreads^(2,3)^. The COVID-19 vaccine was formed through research and innovation to stimulate and increase antibody response to the coronavirus^(4,5)^. This method is effective in preventing the spread of COVID-19, and individuals can help protect their immunity as well as the immunity of others who are vulnerable to COVID-19 infection^(6)^. As a result, an unvaccinated person is more vulnerable to COVID-19 infection, making it difficult for the government and health workers to educate the public about the importance of receiving COVID-19 vaccinations^(3,7)^. Vaccines are effective in reducing COVID-19 infection, according to research in eight countries, so the higher the vaccination rate, the more herd immunity is achieved, and the public’s immunity to COVID-19 infection in each country increases^(8)^. To achieve herd immunity, governments around the world are attempting to accelerate public vaccination.

According to a worldwide survey on the possibility of COVID-19 vaccine acceptance, 48% of the study population was confused about the COVID-19 vaccine and remained unsure whether they would receive the vaccination^(9)^. On September 18, 2021, the global vaccination rate reached 42.9% or 5.88 billion doses of vaccine^(10)^. The results of the Population Census in September 2020 revealed that Indonesia has a population of 270.20 million citizens^(11)^. The Indonesian Ministry of Health aims for a national COVID-19 vaccination coverage of 70% of the total population in each province to achieve herd immunity and effectively control COVID-19 transmission^(12)^. COVID-19 vaccination has only reached 28.02% or around 77.42 million people in Indonesia, with 44.12 million (15.97%) fully vaccinated and 33.29 million (12.05%) partially vaccinated^(10)^. According to the Ministry of Health of the Republic of Indonesia, vaccination was achieved in Banten province for the first dose of 3.64 million people (39.47%) and the second dose of 2.05 million people (22.29 %) out of a target of 9.23 million people^(13)^. According to the Tangerang District Government’s COVID-19 Information & Coordination Center, 869,957 people (34.5%) in the Tangerang District area have received the first dose of the vaccine, and 505,297 people have received the second dose of the vaccine (20%)^(14)^. This statistic is still far short of Tangerang District’s target of 2,514,474 people.

During the peak of the pandemic, vaccine hesitancy can be caused by a variety of factors about vaccinations such as unknown future vaccine effects, doubts about vaccine effectiveness and safety, and potential vaccine side effects led to incorrect threat assessments, maladaptive coping behaviors, and fatal consequences, resulting in vaccine hesitancy cases^(15)^. Misinformation about vaccines and their development process has the potential to raise vaccine distrust in the community, affecting vaccine adherence and attitude toward receiving a vaccination and preventing adequate vaccination levels to achieve herd immunity^(16)^.

A person’s attitude toward receiving a COVID-19 vaccination influences vaccination coverage^(17)^. A study carried out by Islam et al. in Bangladesh revealed that the majority of participants believed that the COVID-19 vaccine could have adverse effects, and half of them did believe that, if everyone in the community took precautions, the COVID-19 pandemic could be avoided without vaccination^(18)^. Several factors influence public attitudes toward COVID-19 vaccination, including knowledge, level of education, and income^(19)^. According to Paul et al., respondents with a lack of background knowledge about COVID-19, low education, and low income have a negative attitude toward the COVID-19 vaccine^(20)^. Furthermore, the opinions of other important people, such as family, friends, and health professionals, influence their willingness to receive vaccines^(21)^. Disinformation spreading on social media can also be an indication of influencing vaccine information to the public, affecting attitudes and adherence to COVID-19 vaccination^(22)^.

Knowledge, perceptions, and attitudes of the community toward COVID-19 are crucial for the government and policymakers to resolve all barriers to vaccine distribution^(18)^. Broad COVID-19 vaccination coverage is critical for COVID-19 control, and it is influenced by the attitudes of those who receive COVID-19 vaccinations^(23)^. Knowledge, education, and financial status all have an impact on attitudes toward receiving COVID-19 vaccinations^(23)^. As a result, understanding these factors is essential, as they influence people’s attitudes toward vaccine acceptance^(23)^. Understanding the population’s diverse needs and the factors that influence public attitudes toward vaccines will aid in the development of evidence-based multilevel interventions to increase global vaccine uptake^(23,24)^. As a result of research, effective interventions to increase vaccine use will be developed, allowing herd immunity to be formed.

## OBJECTIVE

To analyze the factors affecting the community’s attitude toward COVID-19 vaccination in the Tangerang District. The factors investigated include knowledge, education levels, and income.

## METHODS

### Ethical aspects

Ethical consideration was obtained from the Nursing Ethics Committee of the Faculty of Nursing, Universitas Pelita Harapan, whose approval letter is attached to this submission. The study was conducted with ethical principles such as confidentiality, informed consent, autonomy, and justice. To maintain ethical principles, the researcher followed procedures for anonymity and confidentiality in the absence of anonymity, including not requiring the respondent’s name in the questionnaire but rather asking the respondent to write down his initials to maintain the respondent’s confidentiality, and not sharing information about the respondent with other people. Respondents have the right to receive information about research procedures through informed consent, as well as the autonomy to make decisions about whether to participate in research. Meanwhile, to uphold the principle of justice, researchers establish inclusion and exclusion criteria and give respondents equal opportunities to participate in research.

### Study Design, location, and period

The study employs a cross-sectional design in which all variables were measured at the same time. This study was conducted in Tangerang District, and questionnaires were distributed through social media (Facebook, WhatsApp, and Line) in March 2022. The methodology was guided by the STROBE checklist for cross-sectional studies from https://www.equator-network.org/ ^(25)^.

The research information sheet and prior informed consent were attached to the online questionnaire. The researcher provides additional information about the study on the research information sheet to help readers understand its objective, benefit, methodology, ethics, and potential limitations. In addition, there are validation questions for selected participants based on the researcher’s inclusion criteria. These questions include whether the respondent is from Tangerang District, their age range of 18 to 55 and whether they have a source of income. Research participants who meet these inclusion criteria and agree to informed consent will later fill out the informed consent form to be selected as respondents and will proceed to the next session to fill out research questions.

### Population and sample

The population in this study was from Tangerang District and the age ranged from 15 to 59 years old, with a total of 2,211,973 persons in 2020^(26)^. The convenience sampling technique was used, which is a method of selecting respondents based on their availability and ease of obtaining them^(27)^. The Slovin formula was used to obtain 400 respondents. This study’s inclusion criteria are people who live in the Tangerang District, are between the ages of 18 and 55, and have a source of income. People who do not complete the questionnaire and have no income are excluded. The researcher established the age criteria as 18-55 years old because this is the age of adulthood and is included in the productive age of employment according to Ministry Labor regulation No. 02/1995^(28)^.

### Study protocol

A questionnaire with open and closed questions was used in this study as the instrument. The authors used a questionnaire to assess knowledge of COVID-19 vaccination, education level, and income^(21)^ as well as an attitude questionnaire^(22)^. The COVID-19 vaccine knowledge questionnaire consists of 12 questions. A score of more than six indicates high knowledge, while a score of six or less indicates low knowledge. The attitude questionnaire for COVID-19 vaccination includes 12 questions scored on a 4-point Likert scale: 1) strongly agree. 2) agree, 3) disagree, and 4) strongly disagree, with a score indicating a positive attitude (70-100%) and a negative attitude (70Beforer to data collection, the validity and reliability of the knowledge and attitude questionnaires were tested on 30 Tangerang City residents (Cronbach alpha 0.911-0.918). Income is categorized into high, if income is equal to or more than the district minimum wage (≥ IDR 4,262,015), and lw, if income is less than the district minimum wage (< IDR 4,262,015)^(29)^. Education level is categorized into; high consists of a Diploma, Bachelor, Master, and Doctorate; medium consists of Senior High School; low consists of No School, Elementary School, and Junior High School^(30)^.

### Analysis of results and statistics

The collected data are analyzed using univariate and bivariate analytical techniques. Univariate analysis is used to describe each independent (knowledge, education level, and income) and dependent (attitude) variable. Meanwhile, bivariate analysis was employed to determine the correlation between knowledge, education level, and income with the community’s attitudes toward COVID-19 vaccination. In this study, the independent variables were knowledge, level of education, and income. The Chi-square test was used for bivariate analyses. The Statistical Package for the Social Sciences (SPSS) version 28 software is used for the analysis.

## RESULTS

Data were collected from 400 respondents, and all data were complete. Table 1 represents the characteristics of respondents. There were 204 (51%) respondents in adults, 269 (67.25%) males, 194 (48.50%) had high education, 290 (72.50%) had low incomes, and 312 (78%) had high knowledge.

**Table 1:** Characteristics of Respondents (N=400)

Characteristics	n	%
Age		
Adolescence (18-21 years old)	131	32.75
Adult (22-55 years old)	269	67.25
Gender		
Male	206	51.10
Female	194	48.50
Education		
Low	67	16.75
Medium	139	34.75
High	194	48.50
Income		
Low	290	72.50
High	110	27.50
Knowledge		
Low	88	22.00
High	312	78.00

Table 2 shows that most respondents, 306 (76.50%) have a positive attitude while 94 (23.50%) have a negative attitude toward COVID-19 vaccination. According to Table 2, the majority of the 306 respondents with a positive attitude were female (162 respondents) and adults (268 respondents).

**Table 2:** Attitudes of Respondents toward COVID-19 Vaccination (N=400)

Attitude	n	%
Negative	94	23.5
Positive	306	76.50

According to Table 3, 36 (9%) of respondents have low knowledge and a positive attitude toward COVID-19 vaccination, while 270 (67.5%) have high knowledge and a positive attitude toward COVID-19 vaccination. The Chi-square test had a p-value of 0.001, indicating a significant correlation between respondents’ knowledge and attitudes toward COVID-19 vaccination. According to the findings, the value of OR was 9.28 (95% CI: 5.44-15.86), indicating that respondents with high knowledge have a 9.28 times greater chance of a positive attitude than respondents with low knowledge.

**Table 3:** The Correlation between Knowledge and Attitude towards COVID-19 Vaccination (N=400)

Knowledge	Attitude				p value	OR	95 %CI
	Negative		Positive				
	n	%	n	%			
Low	52	13	36	9	0.001	9.28	5.44-15.86
High	42	10.5	270	67.5			

According to Table 4, 34 (8.5%) of respondents have a low education level and a positive attitude toward COVID-19 vaccination, 88 (22%) have a moderate education level and a positive attitude toward COVID-19 vaccination, and 184 (46%) have a high education level and a positive attitude toward COVID-19 vaccination. The Chi-square test produced a p-value of 0.001, indicating that there was a meaningful correlation between respondents’ education level and their attitudes toward COVID-19 vaccination.

**Table 4:** The Correlation between Education Level and Attitude towards COVID-19 Vaccination (N=400)

Education Level	Attitude				p value	OR	95%CI
	Negative		Positive				
	n	%	n	%			
Low	33	8.25	34	8.50	0.001		
Moderate	51	12.75	88	22		0.06	0.03-0.12
High	10	2.50	184	46		0.09	0.05-0.19

The study found that there were 215 (53.75%) respondents with low incomes who had a positive attitude toward COVID-19 vaccination, while 91 (22.75%) respondents with high incomes had a positive attitude toward COVID-19 vaccination (Table 5). The Chi-square test yielded a p-value of 0.094 and an OR of 1.67 (95% CI: 0.95-2.93), indicating that there is no meaningful correlation between respondents’ income and their attitudes toward COVID-19 vaccination, but respondents with higher incomes have a 1.67 times greater chance of being positive than respondents with lower incomes (Table 5).

**Table 5:** The Correlation between Income and Attitude toward COVID-19 Vaccination (N=400)

Income	Attitude				p value	OR	95%CI
	Negative		Positive				
	n	%	n	%			
Low	75	18.75	215	53.75	0.094	1.67	0.95-2.93
High	19	4.75	91	22.75			

## DISCUSSION

Previous vaccination, vaccination beliefs, and attitudes toward COVID-19 vaccination were also discovered to be significant predictors in many countries. The study findings revealed that most respondents have a positive attitude toward COVID-19 vaccination, adults, and males, have higher education, high knowledge, and low incomes. Furthermore, this study discovered a significant relationship between knowledge and attitudes toward COVID-19 vaccination. This demonstrates that a person’s attitude toward receiving a COVID-19 vaccination can be influenced by their knowledge about COVID-19 vaccination; in other words, if someone has a positive attitude toward COVID-19 vaccination, that person will receive it^(31,32)^. According to the findings of Mahmud et al., (2021), People with good knowledge of COVID-19 and COVID-19 vaccines had 22.23 times more chances of accepting the COVID-19 vaccine than people with less knowledge of COVID-19 and COVID-19 vaccines^(33)^. Moreover, People who disagree with the vaccine’s effectiveness and safety are 90% less likely to receive it than those who agree with it^(33)^.

According to the study findings, most respondents had a higher education level, which had a significant correlation with attitudes toward COVID-19 vaccination. This could be related to People who are more educated might be more knowledgeable and concerned about their health and well-being as a result of increased access to information sources, so they have a high level of alertness and awareness to increase body resistance to prevent contracting COVID-19 by vaccination^(11,24)^. Participants with a university degree and students were 21.38 times more likely to receive the COVID-19 vaccine than those with a lower educational qualification^(33)^. More information can lead to well-informed individuals caring about their health and well-being, which can influence participation in life events that affect them, such as COVID-19 vaccination^(18)^.

On the other hand, adult populations with a higher level of education will be more aware of the benefits of preventive health-related problems, such as the COVID-19 pandemic, and will be more open to new health-related information^(34)^. A well-educated person has a more positive attitude and takes preventive measures against COVID-19 transmissions, such as getting vaccinated and adhering to health protocols^(35)^. Increased trust and acceptance of this health protocol is a result of the information obtained, which results in a positive attitude^(36,37)^. Persons with a lower level of education, on the other hand, have low knowledge about COVID-19, so they tend to have a negative attitude and engage in risky behavior to contract the COVID-19 virus by not receiving the COVID-19 vaccination and failing to follow health protocols properly^(35)^.

The study conducted by Mahmud et al., (2021) and Abebe et al., (2021) revealed that males, adults, and those who reside in urban areas, were more likely to accept the vaccine^(33,34)^. This could be because females are more opposed to precautionary action than men^(18)^. Adults, in addition to having higher education, also tend to be exposed to social media more often. Vaccine hesitancy and resistance also are linked to social media misinformation and attitudes toward such vaccine-preventable diseases^(23)^. Furthermore, Tangerang district, where the research is being conducted, is an urban area. The knowledge about COVID-19 vaccination is significantly higher in urban areas than in rural areas^(18)^. This could be because urban areas have better access to information and program outreach than rural areas. In addition, people who had previous experience receiving any type of vaccine were found to have a better understanding of COVID-19 vaccination, resulting in higher vaccine acceptance^(33)^.

According to the study findings, the respondents had a low income, and there was no significant correlation between their attitudes toward COVID-19 vaccination. This shows that not all respondents with a positive attitude toward COVID-19 vaccination have a high income; in other words, respondents with low incomes can have a positive attitude toward COVID-19 vaccination. This can be attributed to the free vaccinations provided by the Indonesian government for the community. A previous study found that income cannot be used to predict respondents’ attitudes toward COVID-19 vaccination^(38)^. Vaccination recipients in low to middle-income countries have the perception and belief that vaccination is effective and safe in preventing disease spread; this is influenced by the dissemination of information about the benefits of vaccination, which increases their knowledge and insight and encourages a positive attitude^(39)^. This positive attitude and a higher acceptance level of the COVID-19 vaccine are influenced by a desire to learn more about the COVID-19 vaccine’s benefits rather than economic conditions^(38,39)^.

The following steps can be taken to increase vaccination coverage: 1) Vaccines must be equitably distributed and equitably, focusing on the most vulnerable individuals; 2) planned mass COVID-19 vaccination programs must take action to address recognized possible obstacles to vaccine acceptance using linguistically and culturally appropriate messages; and 3) Public health officials should build strong COVID-19 vaccine education campaigns utilizing traditional and social media, with a special focus on involving social influencers and target audiences^(40)^. The most effective method of reaching out to communities with COVID-19 information was through evidence-based communication through the use of mass media, an effective policy of providing public health educational campaigns, and outreach activities that were evenly distributed in urban and rural areas^(41,42)^.

### Study limitation

The authors recognize that there were limitations, such as the cross-sectional study cannot infer causality because the temporal relation between the dependent and the independent variable has an unidentified temporal sequence. Furthermore, the authors may be unaware of other factors that may be confounding factors in COVID-19 vaccination attitudes that must be controlled. Furthermore, data collection using online questionnaires is limited to only reaching respondents who have mobile phones or gadgets, and researchers do not have a direct connection with respondents to validate or clarify if necessary.

### Contributions to nursing, health, or public policy

The study findings can be used as input for nurses or health officials to improve community knowledge and attitudes toward COVID-19 vaccination through continued dissemination of information about the benefits and safety of vaccination, particularly for people in rural areas and with low education, direct visits to people with limited access to information to promote a positive attitude, as well as comprehensive campaigns involving health cadres and community leaders. Furthermore, health policymakers can increase vaccination coverage by making vaccination more accessible, instituting mandatory vaccine policies, and launching massive health advertisements in public places or on social media.

## CONCLUSIONS

The study found that most Tangerang District respondents had high education, low income, high knowledge, and a positive attitude. Furthermore, the study discovered a meaningful correlation between respondents’ knowledge and attitudes toward COVID-19 vaccination, with high knowledge having a 9.284 times greater chance of having a positive attitude than low knowledge. However, the study discovered a significant correlation between respondents’ education level and their attitudes toward COVID-19 vaccination, but no correlation between respondents’ income and their attitudes toward COVID-19 vaccination.

According to the findings, health officials should implement immediate socialization programs and disseminate more correct information. Policymakers should consider taking steps to promote sufficient knowledge and positive attitudes toward COVID-19 vaccinations to decrease vaccine hesitancy, which is facilitated and encouraged by media misleading information.
